# Near-Infrared Hyperspectral Imaging to Predict Intact Sweet Tamarind Fruit Quality

**DOI:** 10.3390/foods15142492

**Published:** 2026-07-14

**Authors:** Woranitta Sahachairungrueng, Wayan Dipasasri Aozora, Achiraya Tantinantrakun, Rachit Suwapanich, Saranya Workhwa, Anthony Keith Thompson, Sontisuk Teerachaichayut

**Affiliations:** 1 Department of Food Technology, Faculty of Technology, Khon Kaen University, Khon Kaen 40002, Thailand; wasaha@kku.ac.th; 2 Department of Food Science, School of Food-Industry, King Mongkut’s Institute of Technology Ladkrabang, Bangkok 10520, Thailand; aozoraola@gmail.com (W.D.A.); tantinantrakun.ac@gmail.com (A.T.); 3 Department of Food Process Engineering, School of Food-Industry, King Mongkut’s Institute of Technology Ladkrabang, Bangkok 10520, Thailand; rachit.ch@kmitl.ac.th; 4 Department of Food Technology and Innovation, Faculty of Technology and Engineering, Udon Thani Rajabhat University, Udon Thani 41000, Thailand; saranyaworkhwa@gmail.com; 5 Department of Postharvest Technology, Cranfield University, College Road, Cranfield MK43 0AL, Bedford, UK; keiththompson28@yahoo.com

**Keywords:** calibration, classification, non-destructive, prediction, spectra

## Abstract

The quality of sweet tamarind fruit, as determined by its total soluble solids (TSS), titratable acidity (TA), and TSS/TA ratio, is important for consumer satisfaction. Nondestructive techniques are therefore required to assess the quality of sweet tamarind fruit. This study investigated whether near-infrared hyperspectral imaging (NIR-HSI) in the wavelength range of 935–1720 nm can be used as a non-destructive method to assess TSS, TA, and the TSS/TA ratio of sweet tamarind fruit and to classify it under commercial standards. NIR-HSI-based chemometric and machine-learning modeling was applied for quantification and qualification analyses. Calibration models for determining TSS, TA, and the TSS/TA ratio were developed using partial least squares regression (PLSR) and support vector machine regression (SVMR). A combination of first derivative and SNV spectral pretreatment was optimized to establish an SVMR model for TSS determination. MSC spectral pretreatment was optimized to develop the SVMR model for TA assessment, and the first derivative spectral pretreatment was optimized to establish an SVMR model for the TSS/TA ratio. Correlation coefficients of prediction (R_p_) of 0.959, 0.961 and 0.957 were obtained with root mean square errors of prediction (RMSEP) of 1.102%, 0.369% and 7.850, and a ratio of performance to deviation (RPD) of 3.29, 3.52 and 3.34 for the TSS, TA, and TSS/TA ratio evaluations, respectively. Partial least squares–discriminant analysis (PLS-DA) and support vector machine classification (SVMC) were used for classifying sweet tamarind fruit under a commercial acidity standard (≤4%). The SVMC with SNV spectral pretreatment produced the best prediction results for distinguishing standard and off-standard sweet tamarind fruit with an 82.86% accuracy. NIR HSI can be used to non-destructively predict the quality of tamarind fruit. It can be applied for online sorting to evaluate individual sweet tamarind fruits for grading and quality control in factory environments.

## 1. Introduction

Tamarind (*Tamarindus indica* L.) is a vital leguminous species and a member of the Fabaceae family [1]. Its origin is tropical Africa, but currently the tamarind tree is widely cultivated across tropical and subtropical regions, including India, Pakistan, Thailand, and the Caribbean [2]. There are many tamarind varieties and cultivars, but they are generally classified into sweet and sour depending on their flavor and morphological characteristics [3]. Sweet tamarind is a tropical fruit well-known for its unique taste arising from its balanced sweetness and acidity. It is also nutritionally dense, providing a rich source of essential nutrients and bioactive compounds that contribute to various health benefits [2]. Sweet tamarind is a commercially important tropical fruit [4]. Global exports of fresh sweet tamarind exceeded 1.2 million metric tons in 2024, with a total value of over 2 billion USD [5]. Sweet tamarind is an elongated, pod-like legume comprising three distinct layers: a brittle, light-to-dark brown shell (exocarp); a soft, sticky pulp (mesocarp); and seeds. Upon ripening, the pulp develops a deep brown hue and its signature sweet-and-sour flavors. Inside this pulp are glossy, flattened seeds that range in color from dark brown to ebony [6]. Sweet tamarind predominantly contains tartaric acid, which remains upon ripening and is accompanied by increased sugar levels [7,8]. Tamarind pulp contains low amounts of water, high levels of carbohydrate, protein, and minerals [9]. References [10,11] reported that customer preferences for sweet tamarind depended on fruit maturity as well as its sugar and acid content, which are related to its flavor. Despite high market demand, producers and exporters face a challenge of inconsistent sweetness and acidity. For instance, the levels of sugar and acid are important for producing sauces that use tamarind as a raw material. The fruits’ internal attributes cannot be assessed by conventional visual inspection. Chemical analysis of total soluble solids (TSS) and titratable acidity (TA) is time-consuming, destructive, labor-intensive, and environmentally unfriendly owing to the use of chemicals [12]. Therefore, a non-destructive method for quality analysis would be very useful. Near-infrared spectroscopy (NIRs) combined with chemometrics has been used as a scalable tool for rapid screening in various agricultural applications and in the food supply chain [13,14]. NIRs that can be used in various applications, is a well-suited method to evaluate food, agriculture, and forest products [15,16]. Earlier research reports of its wide application for non-destructive food quality testing, especially for apples [17], mangoes, tangerines, and avocados [18], grapes [19], lychee [20], and passion fruit [21]. However, conventional NIR yields spectral information only at the point of measurement. Therefore, for large fruits, many measurements at various points of each fruit body are needed. Then, this spectral information is averaged to yield representative values. This is difficult to do in a production environment. Previously, conventional NIR was used for sweet tamarind fruit with long pods to predict its internal qualities [10,22]. Also, it was used for pineapples to evaluate their nitrate levels [23]. Various segments of each fruit had to be measured, which was labor- and time-intensive.

NIR can be combined with hyperspectral imaging (HSI) in an advanced method that integrates imaging and spectral techniques into a single system. A three-dimensional data cube is provided by NIR HSI, which can be used to create a spatial map [24,25]. The vibrational behavior of organic molecules provides the complex data obtained from NIR-HSI. It yields information about C-H, C-O, O-H, and C-C groups [26]. Thus, the vibration energy of organic molecules in a sample can be detected using NIR-HSI. This means that the detection of specific chemical groups is possible from the vibrational behavior of each organic molecule. NIR-HSI is therefore a rapid and non-destructive technique that enables online analysis of fruits based on their chemical content.

The absorption characteristics of materials acquired by NIR-HSI and chemometric algorithms can be applied to obtain more useful and understandable data for quantification and qualification models [27,28]. Quality aspects of fruit can be quantitatively predicted by a model employing independent spectral variables and their true chemical composition as dependent variables. Partial least squares regression (PLSR) is suitable for analyzing a large number of independent variables [29]. PLSR uses spectral data as independent variables to predict the chemical content of tested fruit as dependent variables [30]. Support vector machine regression (SVMR) has also been utilized to enhance the quantification of chemical components. As a supervised machine-learning algorithm, SVMR often employs a radial basis function (RBF) kernel to map complex, non-linear data into a higher-dimensional space, enabling the construction of effective decision functions [31,32]. For qualification, partial least squares–discriminant analysis (PLS-DA), which combines regression with linear discrimination [33] and support vector machine classification (SVMC) is a powerful tool that has non-linear classification capabilities. It can be used to resolve complex data relationships by mapping samples into a higher-dimensional space through a kernel function [34]. Previous studies of NIR-HSI have shown that this technique has potential applications for foods and agricultural materials, such as litchi fruit (R_p_^2^ = 0.8594 for anthocyanin content) [35], mangoes (R_p_^2^ = 0.946 for firmness; R_p_^2^ = 0.793 for soluble solids content (SCC); R_p_^2^ = 0.825 for TA) [36], Angelica sinensis (R_p_^2^ = 0.9925 for moisture content and R_p_^2^ = 0.988 for ferulic acid content) [37], apples (R_cv_^2^ = 0.89 for SCC) [38], persimmons (R_p_^2^ = 0.866 for SCC; R_p_^2^= 0.638 for fructose; R_p_^2^ = 0.62 for glucose) [39], hazelnuts (RMSEP = 10 equivalent days of oxidation) [40], tapioca starch (R_p_= 0.996 for predicting the concentration of adulterant) [41], cake (R_p_ = 0.835 for storage time) [42], beef (R^2^ = 0.77 for metabolite profiles) [43], lime (R_p_^2^ = 0.838 for TSS, R_p_^2^ = 0.694 for TA and R_p_^2^ = 0.775 for TSS/TA) [44], eggs (R_p_^2^ = 0.9372 for Pb content; R_p_^2^ = 0.85 for the Haugh unit) [45,46], kiwifruit (95.0%, 97.5%, and 97.5% for classifying storage day of three cultivars) [47], curcumae radix (95.17% for classifying geographic origin authentication with root slices and 99.00% for powders) [48], and mushrooms (97.28% for classifying browning in button mushroom) [49].

The quality of sweet tamarind fruit is indicated by its TSS, TA, and TSS/TA ratio, but these parameters cannot be visually determined. Regulatory requirements control the quality of sweet tamarind fruit in commerce. Acceptable sweet tamarind fruit should have a maximum TA of 4% [50]. Therefore, a nondestructive technique for predicting the TSS, TA, and TSS/TA ratio would be industrially useful. Since sweet tamarind fruits are long pods, representative data along the entire length is necessary for each fruit to establish a model for predicting its chemical content. Measuring spectral NIR-HSI images for each whole fruit is challenging. Also, NIR-HSI-based chemometric and machine-learning modeling for assessing sweet tamarind fruit has not been reported. Therefore, the objective of this research was to test tamarind fruit using NIR-HSI to nondestructively determine its TSS, TA, and TSS/TA ratio and classify standard and off-standard intact fruit for commercial quantification and qualification of its quality.

## 2. Materials and Methods

### 2.1. Tamarind

A total of 115 samples of the sweet tamarind fruit cultivar ‘Si Thong’ grown in Petchabun Province, Thailand, were used in this study. All samples were commercial grade, i.e., without cracks and defects. They were stored in an air-conditioned room at 25 °C for one day before measurements to avoid temperature effects.

### 2.2. Spectral and Spatial Data Acquisition

**Figure 1:**
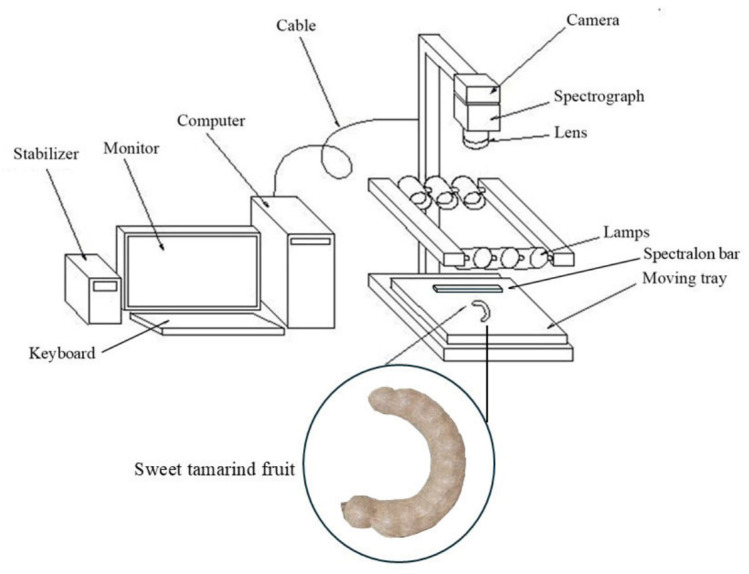
Schematic of the NIR-HSI system for measuring sweet tamarind fruit.

Each whole sweet tamarind fruit was individually scanned using a push-broom hyperspectral camera (Specim FX17e, Spectral Imaging Ltd., Oulu, Finland) in a reflectance mode over the wavelength range of 935–1720 nm and an interval of 3.46 nm (Figure 1). Each fruit was placed on a moving tray with a scanning speed of 15 mm/s, with an exposure time of 5.5 ms. The distance from the sweet tamarind fruit sample to the hyperspectral camera was 20 cm. Each sample was scanned only once. A black reference was obtained when the light source was switched off, and a black cap covered the lens. The white reference was obtained directly from the Spectralon bar. Additionally, each whole sweet tamarind fruit was separated into its pulp, seeds, and shells. All the parts were individually scanned to acquire their spectral information.

### 2.3. Chemical Analyses

After each sweet tamarind fruit was nondestructively scanned using the NIR-HSI system, it was peeled, and the pulp was separated from its seeds and shell. The pulp was used for determining TSS, TA, and the TSS/TA ratio of sweet tamarind fruit. Due to the low moisture content and dry texture of tamarind pulp, direct determinations of TSS and TA were impractical. Therefore, the pulp was first diluted with a known volume of distilled water to prepare a homogeneous extract for analysis. TSS and TA determinations were then performed on the diluted sample, and the obtained values were multiplied by the corresponding dilution factor to calculate the actual TSS and TA values of the original tamarind pulp. Five grams of the pulp was added to 45 g of distilled water and homogenized (IKA, T25 digital ULTRA-TURRAX^®^, Staufen im Breisgau, Germany). It was then passed through a filter cloth to obtain a filtrate. The dilution factor (*D*) was calculated using Equation (1).

(1) $$\mathrm{The}\;\mathrm{dilution}\;\mathrm{factor}\;(D)=\;\frac{weight\;of\;sample+weight\;of\;distilled\;water\;}{weight\;of\;sample}$$

The TSS of the filtrate was measured using a digital refractometer (PR101, Palette Series, Atago Co., Ltd., Tokyo, Japan) as outlined in Ref. [51]. Initially, the TSS of sweet tamarind pulp was calculated as the measured TSS multiplied by *D*. The average value from triplicate measurements was reported.

TA was determined by titration as outlined in Ref. [51]. The filtrate was titrated against 0.1 N sodium hydroxide (NaOH) using phenolphthalein as an indicator until the color changed to pink. The average titer volume used for titration was recorded, and the value of the titratable acidity was calculated using Equation (2) as the percentage of tartaric acid equivalent.

(2) $$\mathrm{TA}\;(\%\;\mathrm{tartaric}\;\mathrm{acid})=\;\frac{0.075\times N_{\mathrm{NaOH}}\times T\times D\times 100\;}{S}$$

where 0.075 is the acid correction factor (CF), *N* is the normality of NaOH, *T* is the volume of NaOH for titration (mL), *D* is the dilution factor, and *S* is the sample mass (g). Each determination for TSS and TA was done three times, and the average value was reported. The TSS/TA ratio of each sample, which was an index of the fruit quality and maturity [52], was calculated.

### 2.4. Data Analysis

**Figure 2:**
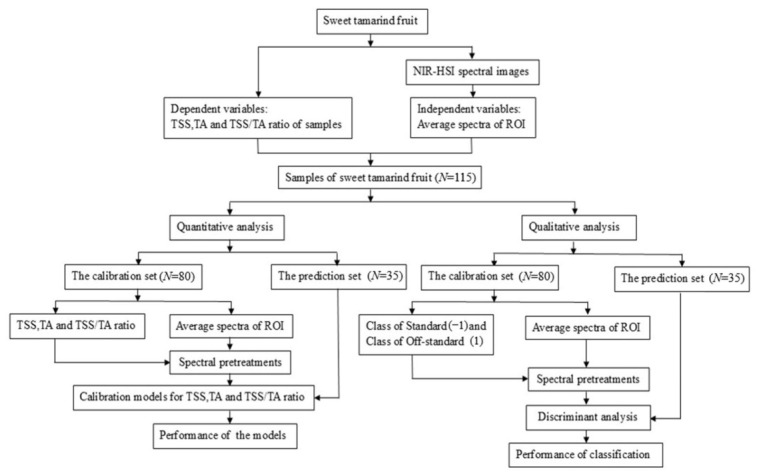
Block diagram for quantitative and qualitative analyses of sweet tamarind fruit.

The procedure for data quantification and qualification analyses of sweet tamarind fruit in this study is presented in the block diagram of Figure 2. The spectral information obtained from NIR-HSI in each scan contained both the spectral data of sweet tamarind and its background. Background data had to be removed to obtain only the spectral data of sweet tamarind fruit. This was done using principal component analysis (PCA). Spectral data represented the effects of the environment, instruments, and sample attributes such as light scattering, natural variability in shape and fruit size, and variations in effective path length [53]. Therefore, algorithmic spectral pre-treatment methods were applied, including Savitzky–Golay smoothing to eliminate noise, the first and second derivatives to correct baseline drifts, the multiplicative scatter correction (MSC), and the standard normal variate (SNV) to correct for signal scattering. The spectral data were varied to identify the optimal spectral pretreatment method for both quantitative and qualitative analyses [54,55].

For quantification analysis, the spectral data of samples in the 935–1720 nm wavelength range were used as independent variables, while TSS, TA, and the TSS/TA ratio were the dependent variables. All samples were divided into calibration and prediction sets. Calibration set samples were used to establish the calibration model and subjected to k-fold cross-validation. By comparison, the optimal spectral pretreatment method was selected based on the lowest root mean square error of cross-validation (RMSECV) and the highest correlation coefficient of cross-validation (R_cv_). Calibration models using the spectral pretreatment methods were developed employing PLSR and SVMR. The best-performing models were selected. The accuracy of the calibration models was determined by testing samples in the prediction set, considering the correlation coefficient of prediction (R_p_), and root mean square error of prediction (RMSEP).

For qualification analysis, samples were categorized into a group of acceptable sweet tamarind fruits (TA ≤ 4%), designated as standard (−1), and a group of unacceptable sweet tamarind fruits (TA > 4%) was designated as off-standard (1). PLS-DA and SVMC were used for classifying groups of sweet tamarind fruit. Samples in the calibration set were used to establish the classification model, while those in the prediction set were employed for testing this model. The optimal spectral pretreatment method was also selected for the best classification. Classification performance was evaluated by considering accuracy (Equation (3)) as the overall percentage of correct classification, specificity (Equation (4)) yielding the percentage of correct classification for the group of acceptable sweet tamarind fruit, sensitivity (Equation (5)) which is the percentage of correct classification for the group of unacceptable sweet tamarind fruit, and error rate (Equation (6)) yielding the overall percentage of incorrect classification [56].

Accuracy (%) = [(*TP* + *TN*)/*Total*] ×100 (3)

Specificity (%) = [*TN*/(*TN + FP*)] ×100 (4)

Sensitivity (%) = [*TP*/*(TP + FN*)] ×100 (5)

Error rate (%) = [(*FP + FN*)/*Total*] ×100 (6)

where *TN*, *TP*, *FN*, and *FP* are the numbers of true negative, true positive, false negative, and false positive samples, respectively. Unscrambler X software (Version 10.4, CAMO Software AS, Oslo, Norway) and UmBio Evince hyperspectral image analysis software (Prediktera Evince, Version 2.7.5, Umeå, Sweden) were used for statistical analysis.

## 3. Results and Discussion

### 3.1. Spectral Data

**Figure 3:**
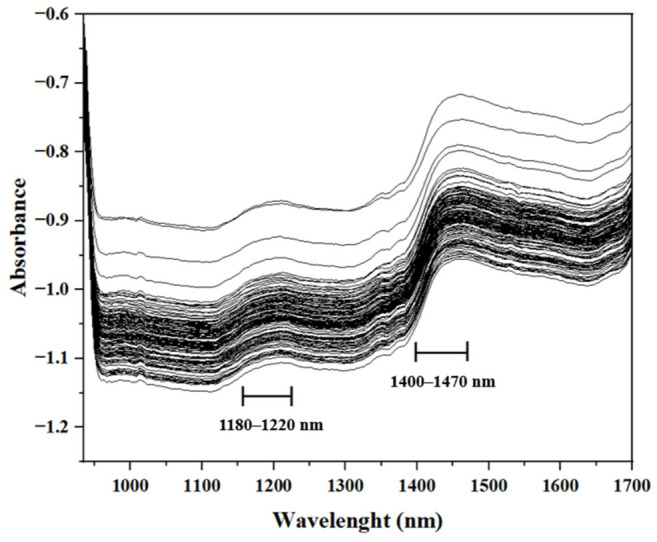
The average original absorbance spectra of sweet tamarind fruit in the wavelength range of 935–1720 nm.

All spectral images in the wavelength range of 935–1720 nm from 115 scans were acquired. The spectral image of each scan was separated into data for the sweet tamarind fruit and background using PCA. The background spectral image was removed, leaving only data for the sweet tamarind fruit. The spectral image of each sweet tamarind fruit was defined as the region of interest (ROI). Since each pixel of the spectral image contained information, the spectral data from each ROI were averaged, as shown in Supplementary Table S1. Average spectra of ROIs (*n* = 115) were acquired as shown in Figure 3, and used as independent variables to establish calibration models in this study. The average spectra of the sweet tamarind fruit showed two broad peaks at around 1180–1220 and 1400–1470 nm. This implied that there were combined peaks of various components in the sweet tamarind fruit. Then, the acquired original absorbance spectra presented overlapping overtone bands of components [57].

**Figure 4:**
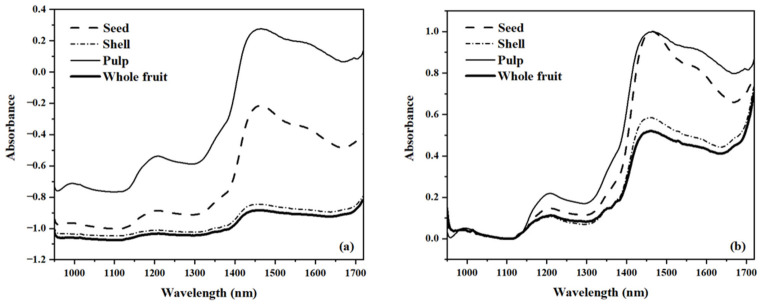
Average absorbance spectra of pulp, seed, shell, and whole fruit of sweet tamarind fruit in the wavelength range of 935–1720 nm: (**a**) original spectra, and (**b**) normalized original spectra.

The average original absorbance spectra of pulp, seeds, shell, and whole fruit in the wavelength range of 935–1720 nm were acquired, as shown in Figure 4a. Baseline drift was observed in the average spectra of pulp, seed, shell, and whole fruit. The main peaks of the average spectra of the shell and whole fruit were not distinct compared to those of the pulp and seed. Therefore, normalization was applied to the spectra (Figure 4b). The normalized absorbance spectra of pulp, seed, shell, and whole fruit showed similar features. The main peaks of these fruit components and whole fruit were also at around 1200 and 1425 nm. Two main peaks presented the absorption band of the C-H second overtone at around 1180–1220 nm and the O-H first overtone at around 1400–1470 nm [58]. The results showed the absorption band of the second stretching peak of the C-H bonds at around 1200 nm, which was associated with the carbohydrate and sugar content [59,60]. An absorption band of the first stretching peak of the O-H bonds appeared at around 1425 nm, which was associated with water [61]. This is in agreement with reports that the carbohydrate content and moisture were high in the pulp, seeds, and shells of tamarind fruit [62,63].

**Figure 5:**
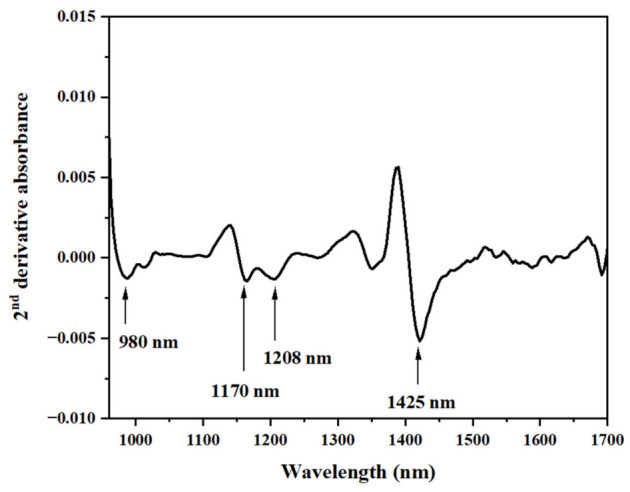
The average second-derivative spectrum of sweet tamarind fruit.

The second-derivative spectral pretreatment was applied to separate the overlapping peaks to determine the individual peaks of the components [64]. Using the second-derivative spectrum enabled the separation of overlapping peaks. Peaks of other components were ascribed to the vibrational behavior of organic molecules, as previously reported by Refs. [65,66]. The average second-derivative spectrum of the sweet tamarind fruit exhibited component peaks at wavelengths of negative peaks, as shown in Figure 5. The negative peaks of the second-derivative spectrum of the sweet tamarind fruit showed wavelengths at the optimum absorbance for specific components such as moisture, crude fiber, and carbohydrates. Peaks at around 980 and 1425 nm corresponded to the O-H stretching overtones of water or moisture [4,61]. The peak at around 1170 nm corresponded to the stretching of the C-H second overtone of the fiber [67]. The peak at around 1208 nm was attributed to the C-H stretching second overtone of carbohydrates [68].

### 3.2. Models for the TSS, TA, and TSS/TA Ratio of Sweet Tamarind Fruit

For quantification, samples were partitioned into a 70:30 ratio for training and testing, which is acceptable for establishing calibration models [69]. Samples were divided into two sets: a calibration set (*n* = 80) and a prediction set (*n* = 35) using the Kennard-Stone algorithm to ensure the calibration set covered the entire variability of the dataset with a uniform distribution of samples in both sets. The characteristics of the dependent variables, TSS, TA, and the TSS/TA ratio of sweet tamarind fruit in the calibration and prediction sets are shown in Table 1. TSS and TA values were reconstructed (measured value × dilution factor *D* = 10); they were not directly measured in this study. The distribution ranges of TSS (59.67–80.33%), TA (0.62–5.55%), and the TSS/TA ratio (11.64–117.04) in the calibration set were wider than those of TSS (64–80.33%), TA (0.71–5.46%), and the TSS/TA ratio (14.31–114.36) in the prediction set. The values of dependent variables in the calibration set coincided with those in the prediction set. The mean values of TSS (75.17%), TA (3.17%), and the TSS/TA ratio (37.46) in the calibration set were similar to those of TSS (75.31%), TA (3.10%), and the TSS/TA ratio (36.42) in the prediction set. The SD of TSS (3.46%), TA (1.26%), and the TSS/TA ratio (28.85) in the calibration set were also similar to those of TSS (3.63%), TA (1.30%), and the TSS/TA ratio (26.25) in the prediction set. The distribution of values in both sets was similar, which is acceptable for establishing and testing the models.

PLSR and SVMR were used to establish calibration models for predicting TSS, TA, and the TSS/TA ratio of sweet tamarind fruit. The spectra of 80 samples in the calibration set were preprocessed using various pretreatment methods. The PLSR and SVMR models were validated using (k = 5)-fold cross-validation to assess the predictive capability of the models from various spectral pretreatments to obtain the optimal conditions for model establishment. Each model acquired from various spectral pretreatments was investigated and compared. The optimum performance of the model was evaluated by considering the lowest RMSECV and the highest R_cv_. By comparison, the optimum model was selected in this study. The prediction accuracy of PLSR and SVMR models was retested using 35 samples in the prediction set. The performance of the PLSR models for predicting TSS, TA, and the TSS/TA ratio of sweet tamarind fruit varied due to differences in the spectral pretreatment methods. This data is shown in Table 2. The smoothing spectral pretreatment method obtained the optimal performance of the PLSR model for TSS (R_cv_ = 0.882, RMSECV = 1.810%). The original spectra with no preprocessing yielded optimal performance for TA (R_cv_ = 0.778, RMSECV = 0.784%). Also, the original spectra with no preprocessing gave optimal performance for the TSS/TA ratio (R_cv_ = 0.819, RMSECV = 16.469).

Spectral data in the calibration set were preprocessed using various pretreatments to establish SVMR models for predicting TSS, TA, and the TSS/TA ratio of sweet tamarind fruit, as shown in Table 3. A combination of the first derivative and SNV spectral pretreatment yielded the optimal performance for the SVMR model for TSS (R_cv_ = 0.966, RMSECV = 1.242%). The MSC spectral pretreatment method showed optimal performance for TA (R_cv_ = 0.973, RMSECV = 0.339%). The SNV spectral pretreatment method presented optimal performance for the TSS/TA ratio (R_cv_ = 0.973, RMSECV = 7.078).

The performance of PLSR and SVMR models for predicting TSS, TA, and the TSS/TA ratio of sweet tamarind fruit was compared in both the calibration and prediction sets. These results are shown in Table 4. They clearly demonstrated that the performance of SVMR models was better than that of PLSR models. The SVMR model for TSS prediction was created using a combination of first derivative and SNV spectral pretreatment, which achieved R_p_ = 0.959 and RMSEP = 1.102%, while the PLSR model created using a smoothing spectral pretreatment method obtained R_p_ = 0.832, RMSEP = 2.021%. The SVMR model for TA prediction developed using the MSC spectral pretreatment method achieved R_p_ = 0.961 and RMSEP = 0.369%, while the PLSR model formulated using the original spectra yielded R_p_ = 0.735, RMSEP = 0.889%. The SVMR model for predicting the TSS/TA ratio created using the SNV spectral pretreatment method achieved R_p_ = 0.957, RMSEP = 7.850, while the PLSR model employing the original spectra obtained an R_p_ = 0.801, RMSEP = 17.925. From these results, it can be concluded that the accuracy and robustness of the SVMR models for predicting TSS, TA, and the TSS/TA ratio in sweet tamarind fruit were better than those of the PLSR models. The combination of first derivative and SNV spectral pretreatment methods provided the best predictive performance for the SVMR model in determining the TSS of sweet tamarind fruit. This could be attributed to their ability to enhance slope variations while suppressing baseline drift, improving spectral resolution, minimizing baseline variations, scattering effects, and amplitude shifts in the spectral data. The MSC spectral pretreatment method was the most effective for improving the predictive performance of the SVMR model in determining the TA of sweet tamarind fruit because it effectively reduced baseline shifts, path length variations, and scattering effects in the spectral data. The SNV spectral pretreatment method yielded the best predictive performance of the SVMR model for predicting the TSS/TA ratio of sweet tamarind fruit because it effectively minimized scattering effects, amplitude shifts, and baseline deviations in the spectral data [70].

**Figure 6:**
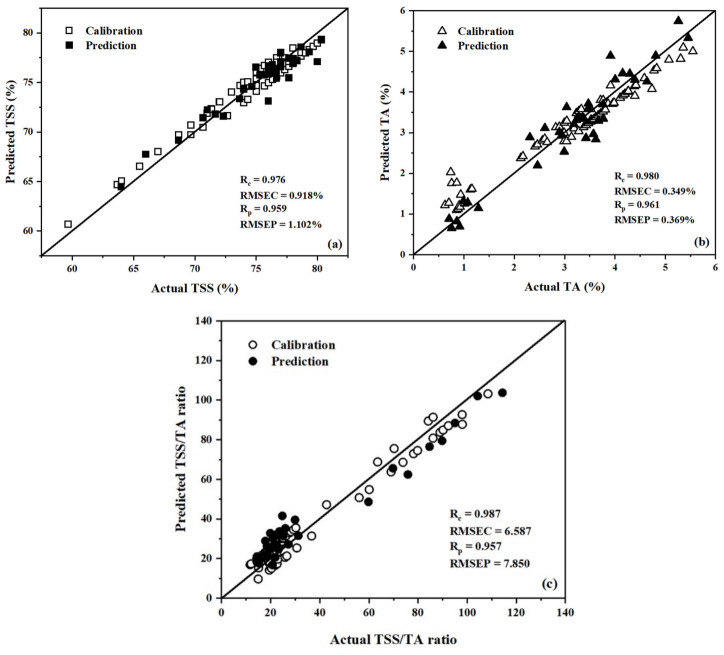
Scatter plots of actual versus predicted values obtained from the SVMR models for predicting quality of sweet tamarind fruit: (**a**) TSS, (**b**) TA, and (**c**) the TSS/TA ratio.

Scatter plots of actual versus predicted values of TSS, TA, and the TSS/TA ratio for the SVMR models in the calibration and prediction sets are presented in Figure 6. Visual distributions of actual and predicted values in the calibration and prediction sets by the SVMR models for TSS (Figure 6a), TA (Figure 6b), and the TSS/TA ratio (Figure 6c) were close to a 45° line, indicating that the performance of the SVMR models was acceptable for predicting the quality of sweet tamarind fruit.

Overall, the results indicate that the NIR-HSI technique has potential applications as a non-destructive, reliable, and accurate technique for predicting tamarind quality attributes.

### 3.3. Classification of Standard and Off-Standard Sweet Tamarind Fruit

For qualitative analyses, the TA values of sweet tamarind fruit (*n* = 115) samples were considered based on an acceptable acidity level (standard, ≤4%) and an unacceptable level (off-standard, >4%). There were 89 acceptable samples (standard, −1) and 26 unacceptable samples (off-standard, 1). The samples were divided into a calibration set (*n* = 80) and a prediction set (*n* = 35). The statistical characteristics of the sample distribution in the calibration and prediction sets are presented in Table 5. The results showed that the mean value of acceptable and unacceptable samples was similar (−0.55 and 0.54, respectively). Also, the SD values of acceptable and unacceptable samples were similar (0.84 and 0.85, respectively), indicating that the distribution of acceptable and unacceptable samples in both sets was similar.

The classification performance based on cross-validation of the models by various spectral pretreatment methods using PLS-DA in the calibration set is shown in Table 6. SNV spectral pretreatment yielded optimal results for establishing a classification model using PLS-DA. It had an 82.50% accuracy, 90.32% specificity, 55.56% sensitivity, and a 17.50% error rate. Table 7 shows the performance based on cross-validation of the classification models by various spectral pretreatment methods using the SVMC calibration set. These results revealed that the SNV spectral pretreatment yielded the optimal classification model with an 88.75% accuracy, 88.71% specificity, 88.89% sensitivity, and an 11.25% error rate.

The classification performance using PLS-DA and SVMC was compared by analyzing the calibration and prediction sets (Table 8). SVMC obtained better classification performance than PLS-DA. Therefore, SVMC with the SNV spectral pretreatment method was selected for classifying groups of standard and off-standard sweet tamarind fruit based on the allowed TA. SNV yielded the best classification performance because it effectively reduced scatter effects, amplitude variations, and baseline deviations [70].

**Figure 7:**
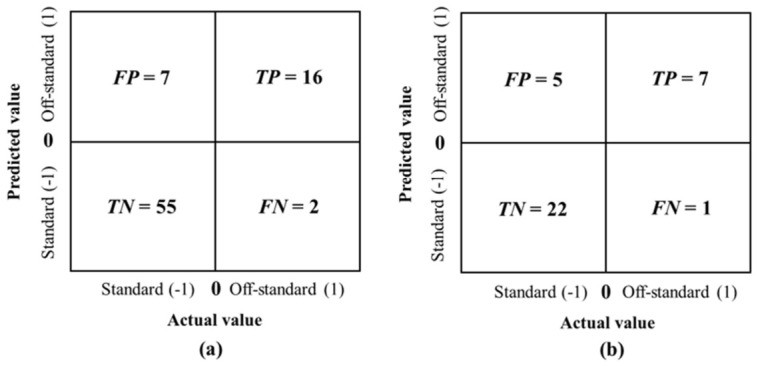
Confusion matrices of actual versus predicted values for standard sweet tamarind fruit (−1) and off-standard sweet tamarind fruit using SVMC (1) in the calibration (**a**), and prediction (**b**) sets.

Figure 7 illustrates the confusion matrices of actual and predicted values using SVMC for classification between standard (−1) and off-standard (1) sweet tamarind fruit. The classification results in the calibration set (Figure 7a) obtained 88.75% (71/80) × 100 accuracy, 88.71% (55/62) × 100 specificity, 88.89% (16/18) × 100 sensitivity, and 11.25% (9/80) × 100 error rate. Classification results for the prediction set (Figure 7b) obtained 82.86% (29/35) × 100 accuracy, 81.48% (22/27) × 100 specificity, 87.5% (7/8) × 100 sensitivity, and a 17.14% (6/35) × 100 error rate. These results indicate that SVMC with the SNV spectral pretreatment method can be used to classify the quality of sweet tamarind fruit based on the commercial standard for TA. Since the same dataset was used for both quantitative and qualitative analyses, only a limited number of off-standard samples were available for the classification study. Consequently, the dataset exhibited an imbalanced distribution between the standard and off-standard classes. Nevertheless, the results demonstrated that the proposed technique still exhibited sufficient potential for classification despite the class imbalance.

This study showed that SVM models achieved better results for both quantification and qualification than PLS models. The relationships between the spectral information in the 935–1720 nm wavelengths and TSS, TA, and the TSS/TA ratio of sweet tamarind fruit appeared somewhat nonlinear. A key advantage of NIR-HSI over conventional NIR spectroscopy is its ability to acquire spectral information over a large area of the sample through hyperspectral imaging, thereby providing a more representative characterization of the whole sample. Because the pulp from each whole long-pod sweet tamarind fruit was used to determine TSS, TA, and the TSS/TA ratio, these parameters were used as the dependent variables. In addition, the average spectrum of ROI was extracted from the hyperspectral image acquired from the entire fruit. Only a single HSI scan was required, enabling rapid measurement while providing a representative spectrum of the whole fruit.

## 4. Conclusions

This study demonstrated the potential of near-infrared hyperspectral imaging (NIR-HSI) techniques for predicting key quality attributes of sweet tamarind fruit, including TSS, TA, and the TSS/TA ratio, as well as for classifying the quality of sweet tamarind fruit under a commercial standard based on TA. For the quantification study, the spectral pretreatment methods were optimized to develop calibration models, including the first derivative combined with SNV as a spectral pretreatment for establishing the SVMR model for TSS, the MSC as a spectral pretreatment for establishing the SVMR model for TA, and the first derivative as a spectral pretreatment for the SVMR model for the TSS/TA ratio. The results clearly indicate that the prediction accuracy of SVMR models is acceptable for use with sweet tamarind fruit. Also, for the qualification study, SVMC with SNV spectral pretreatment was optimized for use in classifying sweet tamarind fruit based on the commercial standard related to TA. It is therefore concluded that NIR-HSI can be developed into a system for quality assessment of sweet tamarind fruit. It is a rapid, non-destructive, accurate, and reliable technique that yields information about a long-pod fruit-like sweet tamarind in a one-pass measurement. The results showed peaks at 980, 1170, 1208, and 1425 nm that could support a feasible industrial multispectral implementation for an online sorting system. It can be commercially developed for grading and classifying individual intact sweet tamarind fruits.

## Figures and Tables

**Table 1:** Characteristics of TSS, TA, and the TSS/TA ratio of sweet tamarind fruit in the calibration and prediction sets.

Attribute	Sample Set	*n* ^4^	Minimum	Maximum	Average	Standard Deviation
TSS ^1^	Calibration	80	59.67%	80.33%	75.17%	3.46%
	Prediction	35	64%	80.33%	75.31%	3.63%
TA ^2^	Calibration	80	0.62%	5.55%	3.17%	1.26%
	Prediction	35	0.71%	5.46%	3.10%	1.30%
TSS/TA ratio ^3^	Calibration	80	11.64	117.04	37.46	28.85
	Prediction	35	14.31	114.36	36.42	26.25

^1^ TA = titratable acidity; ^2^ TSS = total soluble solids; ^3^ TSS/TA ratio = fruit quality and maturity index; ^4^ Number of Samples.

**Table 2:** Performance of PLSR ^1^ models based on different spectral pretreatments for predicting TSS, TA, and the TSS/TA ratio of sweet tamarind fruit.

Preprocessing	Factors	TSS ^2^ (%)		Factors	TA ^3^ (%)		Factors	TSS/TA Ratio ^4^	
		R_cv_ ^5^	RMSECV ^6^ (%)		R_cv_	RMSECV (%)		R_cv_	RMSECV
Original	9	0.859	1.989	**4**	**0.778**	**0.784**	**6**	**0.819**	**16.469**
Smoothing	**7**	**0.882**	**1.810**	4	0.724	0.863	4	0.775	18.115
1st derivative	6	0.846	2.047	4	0.585	1.047	5	0.571	24.265
2nd derivative	6	0.589	3.180	3	0.437	1.145	1	0.505	27.064
MSC ^7^	9	0.814	2.249	3	0.698	0.902	3	0.692	21.690
SNV ^8^	10	0.816	2.245	3	0.707	0.893	3	0.708	21.128
1st derivative + MSC	6	0.814	2.237	3	0.434	1.179	2	0.501	26.953
1st derivative + SNV	6	0.828	2.166	3	0.371	1.222	2	0.496	26.149

^1^ PLSR = partial least squares regression; ^2^ TSS = total soluble solids; ^3^ TA = titratable acidity; ^4^ TSS/TA ratio = fruit quality and maturity index; ^5^ R_cv_ = correlation coefficient of cross-validation; ^6^ RMSECV = root mean square error of cross-validation; ^7^ MSC = multiplicative scatter correction; ^8^ SNV = standard normal variate.

**Table 3:** Performance of SVMR ^1^ models based on different spectral pretreatments for predicting TSS, TA, and the TSS/TA ratio of sweet tamarind fruit.

Preprocessing	Factors		TSS ^2^ (%)		Factors		TA ^3^ (%)		Factors		TSS/TA Ratio ^4^	
	c ^5^	γ ^6^	R_cv_ ^7^	RMSECV ^8^ (%)	c	γ	R_cv_	RMSECV (%)	c	γ	R_cv_	RMSECV
Original	100	0.01	0.856	2.320	1	1	0.945	0.504	1	1	0.919	13.247
Smoothing	100	0.1	0.905	2.132	100	0.1	0.937	0.529	100	0.1	0.924	11.189
1st derivative	10	0.01	0.934	2.024	1	0.01	0.907	0.593	1	0.1	0.903	16.314
2nd derivative	100	0.01	0.933	2.025	1	0.01	0.903	0.700	1	0.01	0.921	12.273
MSC ^9^	100	0.01	0.964	1.265	**10**	**0.01**	**0.973**	**0.339**	10	0.01	0.970	7.421
SNV ^10^	100	0.01	0.937	1.583	10	0.01	0.932	0.904	**1**	**100**	**0.973**	**7.078**
1st derivative + MSC	100	0.1	0.963	1.279	10	0.01	0.966	0.468	10	0.01	0.958	9.344
1st derivative + SNV	**100**	**0.1**	**0.966**	**1.242**	10	0.01	0.971	0.449	10	0.01	0.963	8.699

^1^ SVMR = support vector machine regression; ^2^ TSS = total soluble solids; ^3^ TA = titratable acidity; ^4^ TSS/TA ratio = fruit quality and maturity index; ^5^ c = penalty factor; ^6^ γ = kernel function parameter gamma; ^7^ R_cv_ = correlation coefficient of cross-validation; ^8^ RMSECV = root mean square error of cross-validation; ^9^ MSC = multiplicative scatter correction; ^10^ SNV = standard normal variate.

**Table 4:** PLSR and SVMR methods for predicting TSS, TA, and the TSS/TA ratio in sweet tamarind fruit.

Attribute	Method		Preprocessing	R_c_ ^9^	RMSEC ^10^	R_p_ ^11^	RMSEP ^12^	RPD ^14^
TSS ^1^ (%)	PLSR ^4^		Smoothing	0.888	1.681%	0.832	2.021%	1.80
	Factors = 6							
	**SVMR** ^5^		**1st derivative + SNV** ^8^	**0.976**	**0.918%**	**0.959**	**1.102%**	**3.29**
	**c** ^6^ = **100**	**γ** ^7^ = **0.1**						
TA ^2^ (%)	PLSR		Original	0.845	0.668%	0.735	0.889%	1.46
	Factors = 5							
	**SVMR**		**MSC** ^13^	**0.980**	**0.349%**	**0.961**	**0.369%**	**3.52**
	**c** = **10**	**γ** = **0.01**						
TSS/TA Ratio ^3^	PLSR		Original	0.873	13.947	0.801	17.925	1.46
	Factors = 6							
	**SVMR**		**SNV**	**0.987**	**6.587**	**0.957**	**7.850**	**3.34**
	**c** = **100**	**γ** = **0.01**						

^1^ TSS = total soluble solids; ^2^ TA = titratable acidity; ^3^ TSS/TA ratio = fruit quality and maturity index; ^4^ PLSR = partial least squares regression; ^5^ SVMR = support vector machine regression; ^6^ c = penalty factor; ^7^ γ = kernel function parameter gamma; ^8^ SNV = standard normal variate; ^9^ R_c_ = correlation coefficient of calibration; ^10^ RMSEC = root mean square error of calibration; ^11^ R_p_ = correlation coefficient of prediction; ^12^ RMSEP = root mean square error of prediction; ^13^ MSC = multiplicative scatter correction; ^14^ RPD = ratio of performance to deviation.

**Table 5:** Statistical characteristics of samples: standard (−1) and off-standard (1) in the calibration and prediction sets.

Statistical Parameter	Calibration Set	Prediction Set
*n* ^1^	80	35
Minimum	−1	−1
Maximum	1	1
Average	−0.55	−0.54
Standard deviation	0.84	0.85

^1^ Number of Samples.

**Table 6:** Classification performance using PLS-DA ^1^ by various spectral pretreatment methods in the calibration set.

Preprocessing	Factors	Standard (−1)		Off-Standard (1)		% Accuracy	% Specificity	% Sensitivity	% Error Rate
		*TN* ^2^	*FP* ^3^	*TP* ^4^	*FN* ^5^				
Original	4	58	4	4	14	77.50	93.55	22.22	22.50
Smoothing	5	59	3	6	12	81.25	95.16	33.33	18.75
1st derivative	1	61	1	1	17	77.50	98.39	5.56	22.50
2nd derivative	1	61	1	1	17	77.50	98.39	5.56	22.50
MSC ^6^	4	57	5	7	11	80.00	91.94	38.89	20.00
**SNV** ^7^	**5**	**56**	**6**	**10**	**8**	**82.50**	**90.32**	**55.56**	**17.50**
1st derivative + MSC	9	48	14	5	13	66.25	77.42	27.78	33.75
1st derivative + SNV	1	62	0	1	17	78.75	100.00	5.56	21.25

^1^ PLS-DA = partial least squares–discriminant analysis; ^2^ *TN* = True negative; ^3^ *FP* = False positive; ^4^ *TP* = True positive; ^5^ *FN* = False negative; ^6^ MSC = multiplicative scatter correction; ^7^ SNV = standard normal variate.

**Table 7:** Classification performance using SVMC ^1^ by various spectral pretreatment methods in the calibration set.

Preprocessing	Factors		Standard (−1)		Off-Standard (1)		% Accuracy	% Specificity	% Sensitivity	% Error Rate
	nu ^2^	γ ^3^	*TN* ^4^	*FP* ^5^	*TP* ^6^	*FN* ^7^				
Original	0.255	0.1	23	39	10	8	41.25	37.10	55.56	58.75
Smoothing	0.255	1	37	25	4	14	51.25	59.68	22.22	48.75
1st derivative	0.01	1	45	17	10	8	68.75	72.58	55.56	31.25
2nd derivative	0.01	1	42	20	11	7	66.25	67.74	61.11	33.75
MSC ^8^	0.255	1	56	6	8	10	80.00	90.32	44.44	20.00
**SNV** ^9^	**0.255**	**10**	**55**	**7**	**16**	**2**	**88.75**	**88.71**	**88.89**	**11.25**
1st derivative + MSC	0.01	0.01	31	31	14	4	56.25	50.00	77.78	43.75
1st derivative + SNV	0.01	10	43	19	12	6	68.75	69.35	66.67	31.25

^1^ SVMC = support vector machine classification; ^2^ nu = nu parameter; ^3^ γ = kernel function parameter gamma; ^4^ *TN* = true negative; ^5^ *FP* = false positive; ^6^ *TP* = true positive; ^7^ *FN* = false negative; ^8^ MSC = multiplicative scatter correction; ^9^ SNV = standard normal variate.

**Table 8:** Classification performance using PLS-DA and SVMC in the calibration and prediction sets.

Methods	Data Sets	Preprocessing	Factors		Standard (−1)		Off-Standard (1)		% Accuracy	% Specificity	% Sensitivity	% Error Rate
					*TN* ^1^	*FP* ^2^	*TP* ^3^	*FN* ^4^				
**PLS-DA** ^5^	**Cal** ^7^	**SNV** ^9^	5		56	6	14	4	87.50	90.32	77.78	12.50
	**Pre** ^8^				23	4	4	4	77.14	85.19	50.00	22.86
**SVMC** ^6^	**Cal**	**SNV**	**nu** ^10^	**γ** ^11^	**55**	**7**	**16**	**2**	**88.75**	**88.71**	**88.89**	**11.25**
	**Pre**		**0.255**	**10**	**22**	**5**	**7**	**1**	**82.86**	**81.48**	**87.50**	**17.14**

^1^ *TN* = true negative; ^2^ *FP* = false positive; ^3^ *TP* = true positive; ^4^ *FN* = false negative; ^5^ PLS-DA = partial least squares–discriminant analysis; ^6^ SVMC = support vector machine classification; ^7^ Cal = calibration set; ^8^ Pre = prediction set; ^9^ SNV = standard normal variate; ^10^ nu = nu parameter; ^11^ γ = kernel function parameter gamma.

## Data Availability

The data presented in this study is available on request from the corresponding author.

## Supplementary Materials

The following supporting information can be downloaded at: https://www.mdpi.com/article/10.3390/foods15142492/s1, Table S1: The average spectral data of tamarind fruits.
